# Points to consider when evaluating three-dimensional digital subtraction angiography of intracranial aneurysms and their effects on treatment

**DOI:** 10.3906/sag-2008-134

**Published:** 2021-06-28

**Authors:** İsmail YARDIMCIOĞLU, Yılmaz ÖNAL, Murat VELİOĞLU, Hakkı Muammer KARAKAŞ

**Affiliations:** 1 Department of Radiology, Osmaniye State Hospital, Osmaniye Turkey; 2 Department of Radiology, Fatih Sultan Mehmet Training and Research Hospital, İstanbul Turkey

**Keywords:** Aneurysm, intracranial aneurysm, 3D angiography, digital subtraction angiography, DSA, interventional neuroradiology, endovascular treatment

## Abstract

**Background/aim:**

In this study, we aimed to investigate what should be regarded as potential determinants of treatment strategies when evaluating 3D digital subtraction angiography (DSA) images.

**Material and methods:**

Our inclusion criteria were as follows: (1) presence of at least one intracranial aneurysm demonstrated by conventional angiography, (2) having both 2D and 3D images, and (3) being over 18 years old. First, two-dimensional (2D) and then 3D angiography images of 226 aneurysms of 150 patients were scanned. Morphological characteristics such as size, configurations, relationship with parent artery, baby counts, and other incidental findings were determined.

**Results:**

Of the 226 aneurysms, 11 (4.9%) were only detected on 3D images. Four of these 11 additional aneurysms were believed to be babies of other aneurysms seen in 2D images. Middle cerebral artery (MCA) M1 segment was the most common localization in terms of missed aneurysms. Of the 28 aneurysms located in the communicating segment of the internal carotid artery, the absolute locations of 7 (25%) could not be detected in 2D images or detected in the wrong location. Of the 24 aneurysms located in the ophthalmic segment, the origin of 8 (33%) could not be clearly identified in 2D images. Truncus relationships of MCAs bifurcation/trifurcation aneurysms were seen in 41 of 63 aneurysms (65%) on 2D images, whereas all were confirmed on 3D images. Fenestrations not seen in 2D images were identified in 3D images of 4 patients (3%).

**Conclusion:**

The superiority of 3D images compared to 2D images in determining the morphologic characteristics of intracranial aneurysms has been known for a long time. The contribution of 3D images to the treatment can be summarized as evaluating the parent artery relationship, revealing the number and shapes of aneurysm babies more clearly, detecting fenestrations, and shortening procedure time by finding the correct working angle.

## 1. Introduction

Although intracranial aneurysms are often detected incidentally through the frequent use of medical imaging today, we may encounter the rupture of these aneurysms as life-threatening bleeding. Subarachnoid hemorrhage (SAH) and associated complications such as vasospasm or hydrocephalus are responsible for mortality and morbidity.

The prevalence of cerebral aneurysms was reported as 3.7% in retrospective angiographic studies and 6% in prospective angiographic studies. Approximately 85% of them are seen in the Willis polygon and are more predominant among the female sex [1]. For these reasons, the diagnosis and treatment of aneurysms are crucial.

Although significant advances have been made in the diagnosis of aneurysms, in light of advances in computed tomography (CT) and magnetic resonance imaging (MRI), the gold standard method for diagnosis is digital subtraction angiography (DSA). 

The primary goal of aneurysm treatment is to break the relationship between the aneurysm and regular circulation. Surgical methods have been used as the only treatment option for years. However, with the introduction of coiling since the 1990s, endovascular treatment (EVT) options have gained importance. Still, both techniques have their advantages and disadvantages. Therefore, deciding the right method of treatment is crucial. In order to determine the treatment modality, three-dimensional (3D) DSA imaging is a must. Handicaps of two-dimensional (2D) images such as the inability to localize the aneurysm with absolute accuracy and the inability to thoroughly evaluate the aneurysm sac’s relationship with neighboring vascular structures are known. Many studies have reported that 3D angiography is superior to other imaging methods in detecting and detailing aneurysms [2–5].

This study aims to investigate the sort of effects 3D DSA imaging may have on aneurysm treatment and to determine the points that should be considered when examining 3D images of patients.

## 2. Material and methods

### 2.1. Patients and statistical analysis

The study was carried out with the approval of the Ethics Committee of Fatih Sultan Mehmet Research and Training Hospital (Date and Number: 28.02.2017 – 254). A total of 163 aneurysm patients who underwent conventional cerebral angiography were detected. The information in the hospital database and the radiological images stored in the picture archiving and communication system (PACS) were retrospectively reviewed by two radiologists, one of whom has 15 years of experience in neuroradiology. First, anterior-posterior (AP) and lateral angiograms of 2D DSA and then 3D volume-rendered (VR) images along with rotational angiographic (RA) images were evaluated subsequently. Thirteen patients whose image quality was not optimal due to patient agitation were excluded from the study. Patient sex, age, and hospital admissions, which may affect treatment, as well as the number, localization, size of the aneurysms, neck width, parent artery, and relationship with adjacent arterial structures, were evaluated. 

Statistical analyses were performed using IBM SPSS 22.0 statistics software (Version 17; IBM Corp, Armonk, NY, USA). Percentage (%), number (n), mean ± standard deviation (x̄ ± sd), and median (IQR) were given in descriptive statistics. Pearson’s chi-squared and Fisher’s exact tests were used in the assessment of categorical variables. The distribution of normality of numerical variables was assessed using the Shapiro–Wilk test and Q-Q plots. For comparison of two different groups, we used the independent samples t-test if they show normal distribution, if not we used the Mann–Whitney U test. The comparison of rates was performed with the z-test. P < 0.05 was considered to indicate a significant difference.

### 2.2. Device and materials

All angiographic images were obtained using the Toshiba Infnx-8000V/G5 (Toshiba Medical Systems Corporation, Otawara, Tochigi, Japan) DSA device, and 3D images were processed on the Vitrea Workstation LT (Vital Images, MN, USA). Cerebral angiography procedures were performed under local anesthesia or sedation, depending on the patient’s general status. Five French Avanti + vascular sheaths (Cordis Corporation, Miami Lakes, FL, USA) were placed in the femoral artery under the ultrasonography device’s guidance. The diagnostic angiography procedure began by administering 30–35 IU/kg intraarterial heparin sodium as a routine. Conventional brain angiography was performed by selecting Radiofocus 0.035-inch diagnostic guidewire (Terumo Corporation, Tokyo, Japan), vertebral 135° tempo aqua (Cordis Corporation, Miami Lakes, FL, USA), and Simmons-2 tempo aqua (Cordis Corporation, Miami Lakes, FL, USA) catheters according to patient age and vascular anatomy. 

### 2.3. Angiography technique

Both vertebral arteries and both internal carotid arteries (ICAs) were selectively catheterized separately. Routinely, contrast media (Omnipaque 300 mg iodine/mL, GE Healthcare, Cork, Ireland) injections were administered by using an automatic injector pump (Mark 7 Arterion, Medrad Inc., Indianola, PA, USA) into both vertebral arteries (VA) and ICAs. Injections were delivered as a total of 9 mL from 3 mL/s for a vertebral artery, and a total of 10 mL from 4 mL/s for an ICA. In patients whose anatomy or vascular structure was not suitable for selective catheterization, vertebral artery (VA) injections were applied from the corresponding subclavian artery, and ICA injections were performed from the corresponding common carotid artery by increasing the amount of contrast media. Two-dimensional DSA images were obtained as two frames per second. 

Both AP and lateral angiograms were obtained for vertebral artery and ICA injections. Aneurysms detected in 2D images or arterial systems likely to contain aneurysms were supported by rotational angiography (RA) and 3D images. These images were simultaneously examined during the procedure, and in necessary cases, additional 2D images were obtained by magnifying the aneurysm site from the angle, which most clearly showed the aneurysm. After adjusting the C-arm and patient’s position, RA imaging began with obtaining the mask image, by rotating the device around the patient at 50° per second for an initial rotation of 200°. Then the C-arm returned to its starting position and rotated again to acquire postcontrast images. The final rotation occurred 2 s after contrast media is delivered from the automatic injector pump, and again rotated at 50° per second for a total rotation of 200°. The amount and injection rate of contrast material administered during RA images were 15 mL in total at 2.5 mL/s and 18 mL in total at 3 mL/s for VA and ICA injections, respectively.

The workstation automatically processed RA images. This process took about 20 s following RA, and 3D images can be viewed on the workstation in 512 ×512 ×512 resolution. These high-resolution images allowed better viewing of vascular structures such as the anterior choroidal artery (AChA) and the perforating branches.

It was possible to obtain more apparent and comprehensible views of the 3D images through various manipulations in the workstation (zoom in–zoom out, vertical-sagittal-horizontal examination, 360° rotation in all angles, removing other vascular structures included in the examination).

The 3D images obtained during EVT were evaluated simultaneously, and appropriate working angles were determined, providing great convenience in terms of both time and planning.

## 3. Results

A total of 150 patients with ages ranging from 26 to 77 (mean 53.8) were included. Ninety-two patients were female (61.3%), and 58 were male (38.7%). Among the 150 patients included in the study, 226 aneurysms were detected. While 205 of the detected aneurysms were located in the anterior circulation, 21 were located in the posterior circulation. The most common site of aneurysms was the MCA bifurcation/trifurcation, with 63 aneurysms (Table 1).

**Table 1:** Number of aneurysms according to detailed localization.

Location	Number of aneurysms
Bifurcation/trifurcation of middle cerebral artery	63
Anterior communicating artery	42
Ophthalmic artery	19
Posterior communicating artery	18
Distal anterior cerebral artery	16
M1 segment of middle cerebral artery	16
Anterior choroidal artery	10
Apex of internal carotid artery	9
Apex of basilar artery	6
Cavernous segment of internal carotid artery	6
Posterior cerebral artery	5
Superior cerebellar artery	5
Paraophtalmic segment of internal carotid artery	5
Posterior inferior cerebellar artery	3
Vertebrobasilar junction	2
M2 segment of middle cerebral artery	1

We detected one aneurysm in 106 patients (70.7%), two aneurysms in 22 patients, three aneurysms in 14 patients, four aneurysms in 6 patients, and five aneurysms in two patients.

The mean maximal diameter of the aneurysms was 6 mm (range: 1.1–47 mm), while the mean neck size was 3.9 mm (range: 0.9–13.1 mm). The two giant aneurysms that were detected were both localized in the ICA paraophthalmic segment.

A total of 120 aneurysms (53%) were detected among the 84 patients (56%) without SAH history, while a total of 106 aneurysms (47%) were detected among the 66 patients (44%) with SAH history. In addition, the aneurysms causing subarachnoid hemorrhage were larger than other aneurysms (7.4 mm (6.5–9.4 mm)) (p < 0.001).

**Figure 1:**
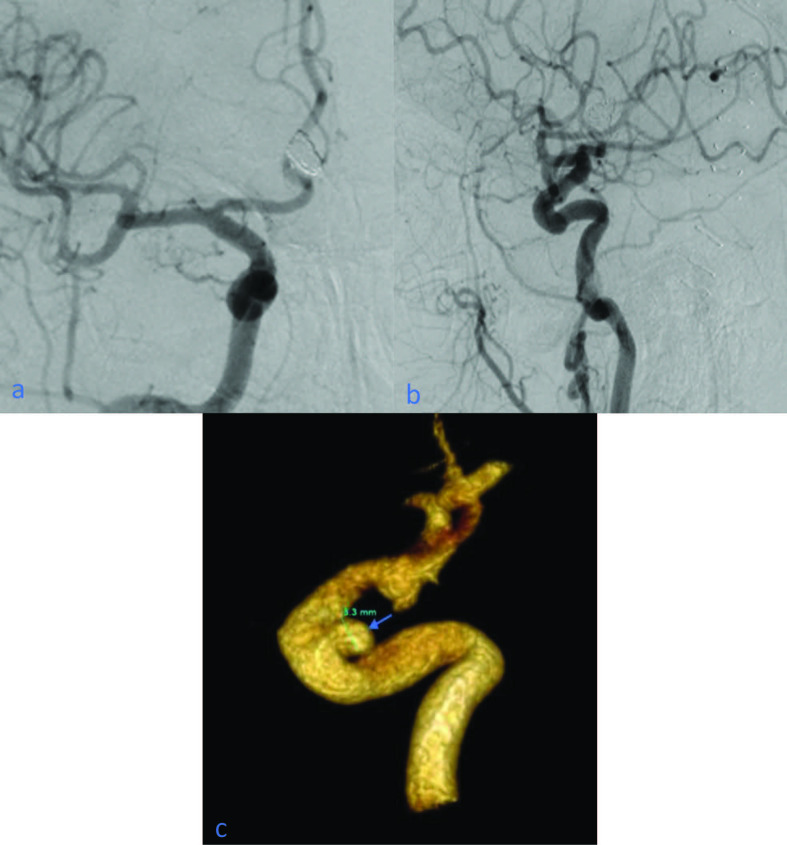
(a, b) Suspected aneurysm appearance is not detected on AP and lateral angiograms; (c) 3.3 mm ICA cavernous aneurysm is detected in the 3D image (arrow).

A total of 11 aneurysms (4.9%) of our study could not be detected on 2D DSA and could only be identified on 3D images (p < 0.001). Six of them were MCA M1 segment aneurysms (55%), two were MCA bifurcation/trifurcation aneurysms (18%), one was an AChA aneurysm (9%), one was an ICA cavernous segment aneurysm (9%), and one was an ophthalmic artery aneurysm (9%), according to localization (Figures 1a–1c). MCA M1 segment was the most common localization in terms of missed aneurysms (p < 0.005). Regarding the comparison of maximal diameter of aneurysms, the mean size of these hidden aneurysms was smaller than apparent aneurysms (2.1 mm (2–2.4 mm)) (p < 0.001).

**Figure 2:**
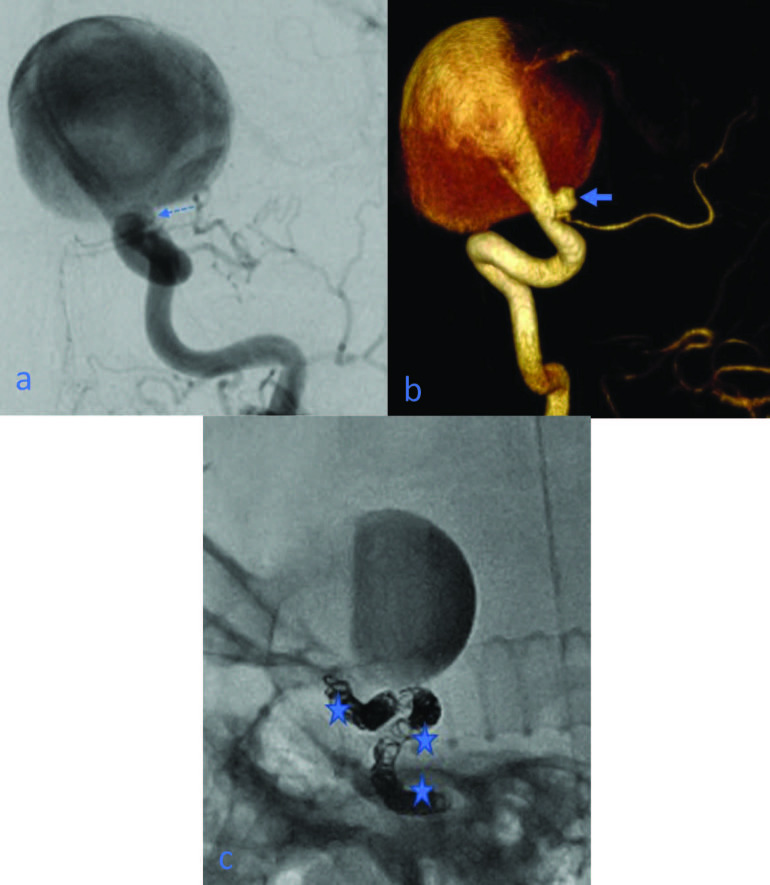
(a) A giant aneurysm is seen in the ICA communicating segment, and due to intraaneurysm contrast stagnation, inadequate ACA and MCA filling is observed in ipsilateral injections. Excessive filling from the contour observed in the inferior portion of the aneurysm was initially evaluated as a baby of the aneurysm (dashed arrow); (b) This structure appears to be an additional ophthalmic artery aneurysm in the 3D image (bold arrow); (c) Intraaneurysm contrast suspension is seen in the follow-up image after parent artery occlusion with coils (stars).

Four of the hidden aneurysms detected only in 3D images were considered babies of other aneurysms. However, when 3D images were examined, it was determined that there were millimeter-sized aneurysms located in close proximity, forming a baby appearance due to superposition. One of these was an AChA aneurysm, one was an ophthalmic artery aneurysm, and two were MCA M1 segment aneurysms (Figures 2a–2c).

**Figure 3:**
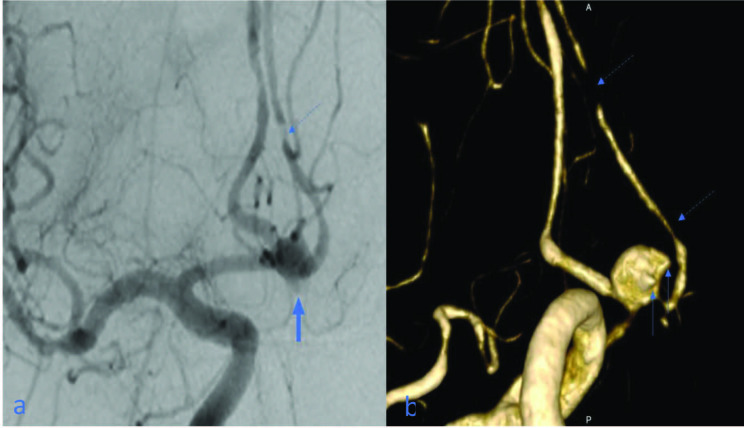
(a) Anterior communicating artery (ACoA) aneurysm is seen in anterior-posterior angiogram, lobulation compatible with baby is seen in the inferior contour of the aneurysm (bold arrow); (b) two babies are seen in the 3D image (arrows); (a, b) critical stenosis in the left ACA A2 segment become prominent in the 3D image (dashed arrows).

In terms of the rupture risk of aneurysms, it is essential to determine the shapes and number of babies. Of the 90 aneurysms in our study which were regarded as not having babies according to 2D DSA, were found to have 0.4 (mean) babies (range: 0–2) per aneurysm, 66 aneurysms thought to have one baby had 1.6 (mean) babies (range: 0–3), 51 aneurysms thought to have two babies had 2.6 (mean) babies (range: 1–4), and 19 aneurysms thought to have three babies were found to have 4.1 (mean) babies (range: 2–5) (Table 2) (Figures 3a and 3b). In terms of rupture risk, aneurysms having three or more babies were clearly associated with subarachnoid hemorrhage (p < 0.001).

**Table 2:** Mean 3D equivalents of aneurysms with babies detected in 2D images.

	Two-dimensional imaging	Three-dimensional imaging
Mean number of babies	0	0.4
	1	1.6
	2	2.6
	3	4.1

**Figure 4:**
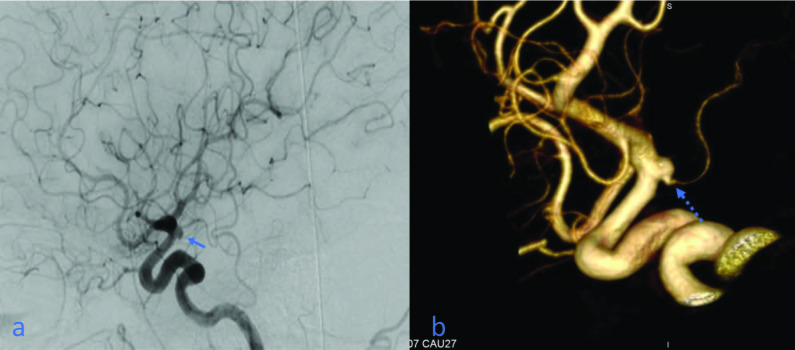
(a) Aneurysm is observed in the communicating segment in the lateral angiogram (arrow), but its origin cannot be detected; (b) In the 3D image, it is observed that the anterior choroidal artery originates from the aneurysm neck region.

In aneurysms located in the ICA communicating segment, it is essential to determine the relationship of the aneurysm with the posterior communicating artery (PCoA) and AChA. In our study, the origins of 7 (25%) of the 28 aneurysms located in the ICA communicating segment were either unidentified or misidentified in the wrong location in 2D images. Two aneurysms believed to be PCoA aneurysms in 2D images were actually AChA aneurysms. Of the five undetermined aneurysms on 2D images, three were PCoA and two were AChA (Figures 4a and 4b).

Another critical localization of the ICA is the ophthalmic segment. In 8 (33%) of the 24 aneurysms located in the ophthalmic segment, the relationship with the ophthalmic artery could not be determined. For the remaining 16 aneurysms, 12 were found to be related to the ophthalmic artery, while four were considered unrelated. Evaluation of 3D images revealed that seven of the undetermined aneurysms were ophthalmic artery aneurysms, and one was a paraopthalmic aneurysm. 

**Figure 5:**
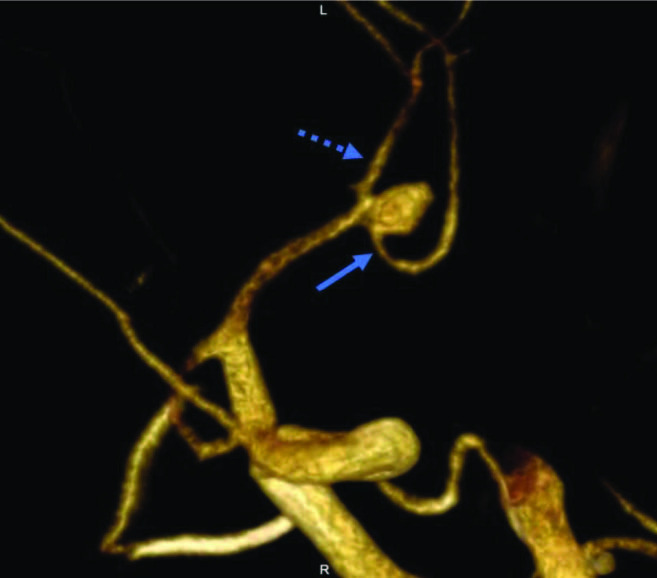
Three-dimensional image shows that a middle cerebral artery aneurysm appears originating from the inferior truncus (arrow), which is unrelated to superior truncus (dashed arrow).

When the 63 MCA bifurcation/trifurcation aneurysms were evaluated with only 2D images, truncus relationships could be accurately confirmed in 41 (65%) aneurysms. Of the remaining 22 (35%) aneurysms, truncus relationships could not be identified or were misidentified in 2D images. When the aneurysms detected in 2D angiograms were examined in 3D images, it was observed that five had a relationship with only the superior truncus, and four had a relationship with only the inferior truncus (Figure 5). One MCA trifurcation aneurysm was found to be related to the superior and middle truncus but was unrelated to the inferior truncus. According to the results, of the 63 MCA bifurcation/trifurcation aneurysms, 10 (16%) were unrelated to at least one truncus. The remaining 53 (84%) MCA bifurcation/trifurcation aneurysms were observed to be related to all trunci, according to 3D volume-rendered images. 

**Figure 6:**
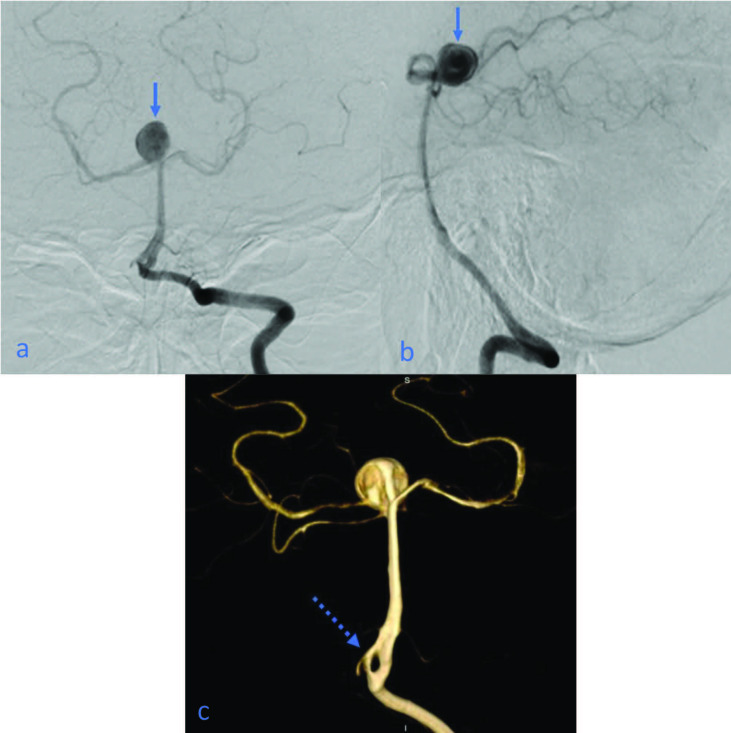
(a, b) With injections to the left vertebral artery, aneurysm is seen at the basilar apex (arrows); (c) fenestration is detected proximal to the basilar artery in 3D images (dashed arrow).

In four patients (3%), significant fenestrations were noticed in 3D images. Two of these (50%) were proximal to the basilar artery, one (25%) was at the vertebrobasilar junction, and one (25%) was at the ACA A1-A2 junction (Figures 6a–6c).

## 4. Discussion

In this study, we investigated the effects of 3D DSA imaging on intracranial aneurysm treatment. Previous studies in the literature have shown that 2D DSA imaging is considerably less sensitive than 3D DSA images in detecting aneurysms [2–5]. In a 2012 study by Küçükay et al. [6] conducted on 122 patients with SAH history, the number of aneurysms missed in 2D images was 15%. Another 2012 study by Wong et al. [7] reported this rate as 7%. In our study, a total of 11 (5%) aneurysms were undetected in 2D DSA and could only be determined in 3D images. This rate was found lower compared to the rates reported in the literature. On the other hand, it was observed in our study that all ACA and vertebrobasilar system aneurysms were completely detected through 2D images. However, 3D images should still be taken for these localizations. Previous studies have detected multiple aneurysms in rates varying from 10% to 30% [8,9]. In our study, multiple aneurysms were detected in 44 patients (29.3%). Due to the complex anatomy of the ICA and MCA, it was understood that millimetric aneurysms in these locations could be overlooked in 2D but could be easily identified in 3D images. In patients with more than one aneurysm, especially in ICA aneurysms, the detection of additional millimetric aneurysms shifts the treatment towards flow-diverting stents rather than surgical approach or coil embolization. This is because the presence of multiple aneurysms requires separate intervention for surgical clipping and coiling, while flow-diverting devices usually allow treatment with a single stent.

The literature reports varying rates for the incidence of aneurysm, according to localization. In a study of 212 patients with <10 mm incidentally detected aneurysms, the most common localization was the MCA with 27% [10]. In our study, MCA also was the most common localization with 80 aneurysms (35%). The low rate of posterior system aneurysms in our study was attributed to the fact that a significant portion of our patients applied to the hospital with SAH since posterior system aneurysms have higher rupture risk and mortality risk in the case of rupture [11–13]. 

It is crucial to determine the number and shapes of babies in terms of rupture risk. 3D images are particularly useful, especially in detecting bleeding aneurysms or determining incidental aneurysms with a high risk of rupture that requires early intervention. In cases involving a curve such as the ICA or MCA, or when the aneurysm cannot be clearly distinguished due to superposition, the evaluation of babies is confusing in 2D images and may yield incorrect results. The number of babies and their structure can be more clearly detected in 3D images. In aneurysms with a high number of babies or wide neck, even if a flow-diverting stent is being considered, placing a few coils inside the sac beforehand increases the rate of total closure and may decrease the risk of recurrent bleeding.

2D images are inadequate in determining the origins of ICA aneurysms. In aneurysms with localization accurately confirmed by 3D images, different treatment alternatives should be considered. In PCoA aneurysms, closure of the PCoA is generally not considered to pose a problem in the presence of well-developed and patent P1 segments. Therefore, while most methods can be alternatives in PCoA aneurysms, this is not the case for aneurysms of the closely neighboring AChA, which carries serious clinical significance. Its closure during treatment may result in hemiplegia or hemianopsia. In case of localization in the dominant side, speech disorder may be added to the clinical picture. In a 2008 study of 53 patients by Cho et al. [14], the rate of infarction developing after surgical clipping of AChA was 22%, and this rate was higher in previously unruptured and <5 mm aneurysms. Srinivasan et al. [15] conducted a study in 2017, in which 18 flow-diverting stents were performed on 18 AChA aneurysms and within the mean 19-month follow-up period, AChA remained patent in all patients. Therefore, during surgery or aneurysm treatment procedures with coil embolization, it is desired that AChA is preserved to prevent ischemic complications due to AChA occlusion. In order to avoid all of these risks, in recent years, the safe and frequently used method of flow-diverting stent is preferred. In this manner, thrombosis of the aneurysm can be ensured, sustaining the flow of AChA.

A similar situation exists regarding aneurysms associated with the ophthalmic artery. Coil embolization or clipping procedures lead to potential vision loss in ophthalmic artery aneurysms. According to the literature, the incidence of new-onset vision loss after ophthalmic artery occlusion is between 4.8% and 7.5%, and 5% after coil embolization, with similar outcomes [16,17]. In these patients, flow-diverting stenting may be considered to be the first choice in treatment. In paraophthalmic aneurysms without ophthalmic artery involvement, coiling or clipping may be the first choice.

While 2D images may be inadequate in determining the origins of MCA bifurcation/trifurcation aneurysms and their relationships with the inferior and superior trunci, it has been established that 3D images are particularly useful in this regard [18]. In our study, the details of 22 (35%) of the 63 MCA bifurcation/trifurcation aneurysms could not be detected. Similar to ICA anatomy, 3D images must be examined in order to detail the anatomy and aneurysms of this region. In this manner, additional information can be obtained in terms of determining the patient’s treatment. Although there is a general tendency towards surgery in MCA aneurysms, those with narrow necks and those that can be applied coil embolization with truncus preservation must be differentiated. When the 3D images of MCA bifurcation/trifurcation aneurysms were examined, it was observed that EVT or coil embolization with truncus preservation could be applied to many of the aneurysms. Thus, less invasive methods could be recommended to patients rather than surgical methods.

Fenestrations are easily detected in 3D images, and most cannot be identified in 2D images. In one study, fenestration was detected in 56 (27%) of the 208 patients, and ACoA was the most common localization as 70% [19]. In another study, 11 of the 12 cases were found to have ACoA fenestration, which could not be detected with 2D images but could only be detected with 3D images [20]. In our study, we identified critical fenestrations in four (3%) of our patients. The detection of fenestration is vital since they complicate endovascular procedures and increase the rate of complications. If vascular anatomy is suitable, contralateral catheterization in EVT may be the considered approach to the aneurysm. However, in cases where this is not possible, surgical intervention is required.

2D images alone are insufficient in evaluating the neck of the aneurysm. In a 2001 study by Anxionnat et al. [3], aneurysm neck could be clearly evaluated in only four of the 22 patients using 2D DSA images. 3D images are vital in clearly visualizing the neck region of the aneurysm. As established in the literature, larger than normal measurement of neck width is a handicap of 3D images. The literature has demonstrated that neck width measurements are calculated larger than normal in 3D images [3,7,21,22]. In order to accurately evaluate the neck region, it is thought that taking measurements from specially taken 2D images in the angle detected in 3D images and in which the neck region is most clearly visualized will yield the most accurate result. In addition to evaluating the neck, it is also important to determine the correct working angles during EVT. It is necessary to use the optimal working angles for reasons such as visualizing the neck of the aneurysm, preventing the superposition of neighboring arteries, or observing the outlet points of the vessels originating from the aneurysm sac. It is not easy to find the working region in 2D images, especially in complex cases, and may require additional imaging. 3D DSA imaging also eliminates this problem. In order to determine the correct working angle, 3D images must be examined in detail, and the angiography machine must be brought to the appropriate angle. Therefore, the operating time, the radiation exposure of the patient and personnel, and the amount of contrast media that is used are reduced.

The lack of depth in 2D images and the superposition of other arterial structures make it challenging to evaluate the shape of the aneurysm. As a result, knowledge of important parameters such as the contours and shape of the aneurysm is significant in terms of choosing the procedures for treatment. 3D images provide valuable information, especially regarding which aneurysm is bleeding in patients with multiple aneurysms.

On the other hand, 3D images are brought to surgical position and serve as a guide for surgeons, predicting what sort of anatomy the surgeon will encounter during the intraoperative period [5,16].

In this study, we aimed to emphasize the importance of 3D DSA imaging before, during, and after the treatment of an aneurysm. However, our study had some limitations. Firstly, there was no comparison of cross-sectional methods, such as CT and MRI, which provide 3D images. There was no participation of a neurosurgeon specialist; therefore, we evaluated surgical treatment concepts using our own experiences. In addition, due to the retrospective nature of the study, not all treatments considered in patients were applied. Further prospective studies conducted together with surgeons will provide more accurate and objective results.

## 5. Conclusion

The definite location, neck structure, evaluation of babies, and anatomic neighbors of aneurysms are absolutely determined with 3D DSA images. In addition, 3D images are essential in terms of detecting hidden accompanying aneurysms and accurately evaluating ICA and MCA aneurysms, which are difficult to evaluate using 2D images. It is necessary to differentiate AChA aneurysms and preserve them in treatment when possible. Fenestrations, which are significant when choosing the main treatment method, must be revealed during the diagnostic phase. It should be noted that the working angles, especially during EVT procedures, can be quickly recognized using 3D images, and accordingly, reducing both radiation exposure and the amount of contrast medium used. All of this important information can be readily accessed with 3D DSA and can be used to guide treatment.

## Funding

This study did not receive any specific funding.

## Informed consent

Informed consent was not required in this study. The study was carried out with the approval of the Ethics Committee of Fatih Sultan Mehmet Research and Training Hospital (Date and Number: 28.02.2017 – 254).
